# Supplementary material for: SFPQ Promotes Homologous Recombination via mRNA Stabilization of RAD51 and Its Paralogs
Source: bioRxiv. 2025 Sep 8:2025.09.08.674956. Preprint. [Version 1] doi: 10.1101/2025.09.08.674956 (PMC12439991; doi:10.1101/2025.09.08.674956)
Supplement: 2 [file NIHPP2025.09.08.674956v1-supplement-2.pdf]

bioRxiv preprint doi: https://doi.org/10.1101/2025.09.08.674956; this version posted September 8, 2025. The copyright holder for this preprint (which was not certified by peer review) is the author/funder, who has granted bioRxiv a license to display the preprint in perpetuity. It is made available under aCC-BY 4.0 International license.

Supplementary Figures:

Figure S1: Genotoxic stress elevates SFPQ intensity and foci formation per cell

(A) (Top) SFPQ mean intensity: Violin plots (with embedded boxplots) show the single-cell distribution of nuclear SFPQ mean fluorescence intensity in DIvA U2OS cells under no break (untreated) and break (4-hydroxytamoxifen, 4-OHT) conditions. Each dot is one nucleus; boxplots denote median and interquartile range. Cell-cycle phase (G1, S, G2) was assigned per cell using EdU incorporation (green) and DAPI DNA content (blue).
(Bottom) SFPQ foci per cell: Violin plots (with embedded boxplots) show the number of SFPQ nuclear foci per cell under the same conditions and cell-cycle stratification.
Quantification: Cells were left untreated or treated with 4-OHT to induce AsiSI-mediated DSBs, then stained for SFPQ (cyan), EdU, and DAPI. Images were analyzed in Cell Profiler to segment nuclei, call SFPQ foci, compute per-nucleus mean intensity and foci counts, and assigned cell-cycle stage from EdU/DAPI features.

(B) Non–pre-extracted immunofluorescence staining of pATM and SFPQ in DIvA U2OS cells with or without DSB induction. Cells were left untreated or treated with 4-hydroxytamoxifen (4-OHT) to induce AsiSI-mediated DSBs and stained for DNA (DAPI, blue), EdU incorporation (green), phosphorylated ATM (pATM, magenta), and SFPQ (cyan). Images were acquired without cytoskeletal (CSK) pre-extraction to visualize total nuclear staining patterns. Merged images show nuclear co-localization of pATM and SFPQ signals in the presence and absence of DNA damage.

Figure S2: SFPQ knockdown reduces RAD51 expression independently of p53

(A) Differential expression analysis of mRNA-seq data comparing DSB versus no-DSB conditions in siNTC-treated DIvA U2OS cells (n=3 biological replicates). Mean $\log_2$ fold change for the same targets is shown as Fig 3A. No significant expression differences were detected for these targets upon DSB induction in control cells.

(B) ChIP-seq data showing SFPQ abundance at sites upstream and downstream of RAD51-paralog genes both without (noDSB) or with (+DSB) 4 hours of DSB induction. Data displayed is the average signal across all 6 RAD51 paralogs.

(C) mRNA-seq $\log_2$ fold changes of transcript expression of the indicated gene or GO category in DIvA U2OS cells treated with siSFPQ compared to siNTC control for 72 hours in the absence of DSBs. Data represent the mean of three biological replicates. Individual p-values were adjusted for multiple comparisons. Aggregate p-values were combined by Fisher's method.

(D) Western blot of DIvA U2OS cells treated with siSFPQ with or without p53 inhibition by PFT-α (30 μM) for 24 hours. Lysates were blotted for SFPQ, HSP70, MDM2 and RAD51.

(E) (Left) Western blot of p53-null K562 cells treated with siSFPQ. Total protein staining is shown as a loading control.

bioRxiv preprint doi: https://doi.org/10.1101/2025.09.08.674956; this version posted September 8, 2025. The copyright holder for this preprint (which was not certified by peer review) is the author/funder, who has granted bioRxiv a license to display the preprint in perpetuity. It is made available under aCC-BY 4.0 International license.

(Right) Quantification of SFPQ and RAD51 normalized band intensities relative to total protein is graphed.

Figure S3: Post-transcriptional regulation of RAD51 by SFPQ.

(A) Biological replicates of RAD51 protein stability assays. DIvA U2OS cells were transfected with non-targeting control (siNTC) or SFPQ-targeting (siSFPQ) siRNA for 72 hours, followed by treatment with cycloheximide (CHX) alone or CHX plus carfilzomib (Carf). Lysates were collected at the indicated time points (0, 2, and 4 hours) and RAD51 abundance was measured by Western blot, normalized to total protein.

Figure S4: Correlation between SFPQ and RAD51 expression.

(A) Scatterplot showing RAD51 expression (TPM) as a function of SFPQ expression across 1684 cell lines available in Depmap. (Pearson r = 0.625; $p = 6.9\times10^{-1}$[illegible]).

Figure S1

bioRxiv preprint doi: https://doi.org/10.1101/2025.09.08.674956; this version posted September 8, 2025. The copyright holder for this preprint (which was not certified by peer review) is the author/funder, who has granted bioRxiv a license to display the preprint in perpetuity. It is made available under aCC-BY 4.0 International license.

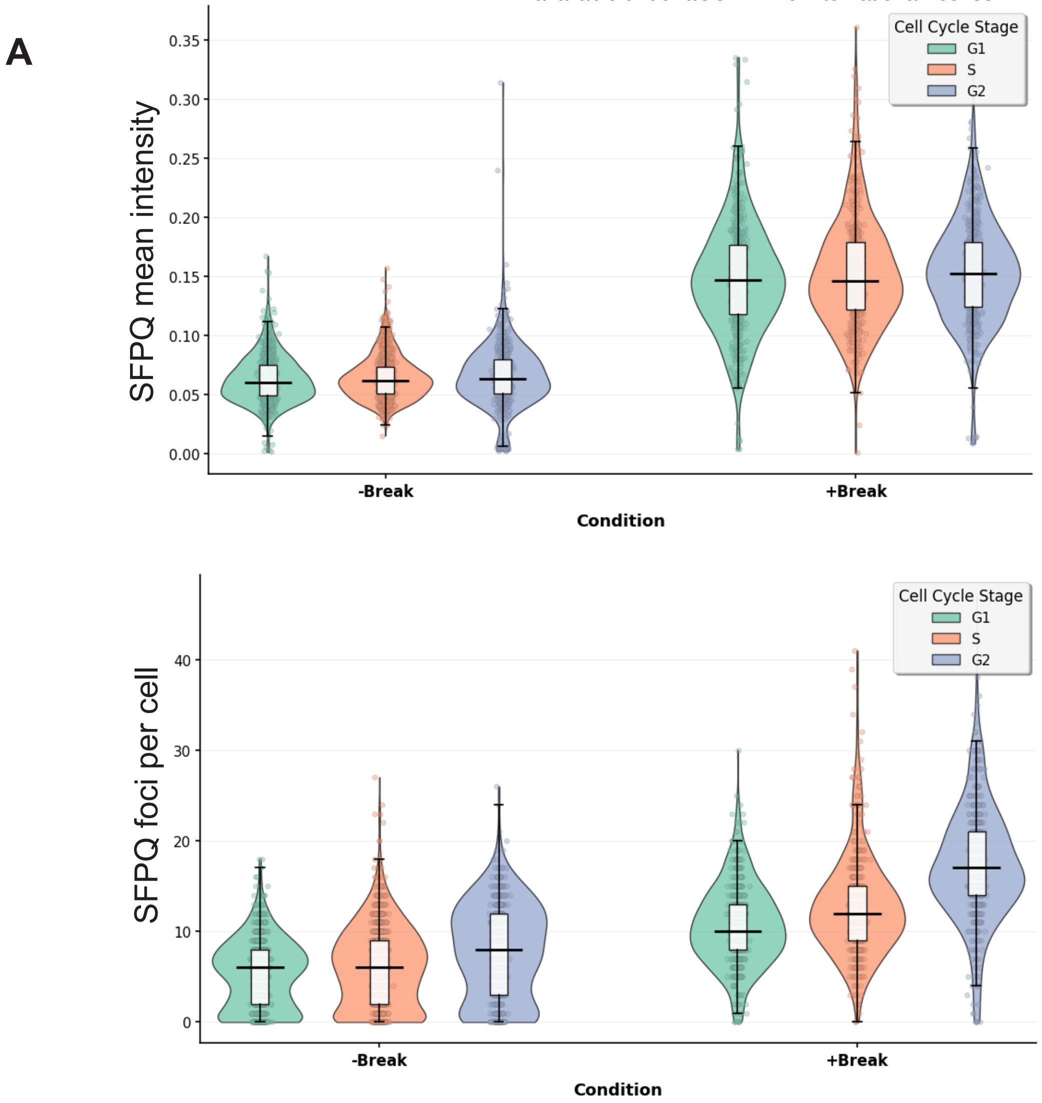

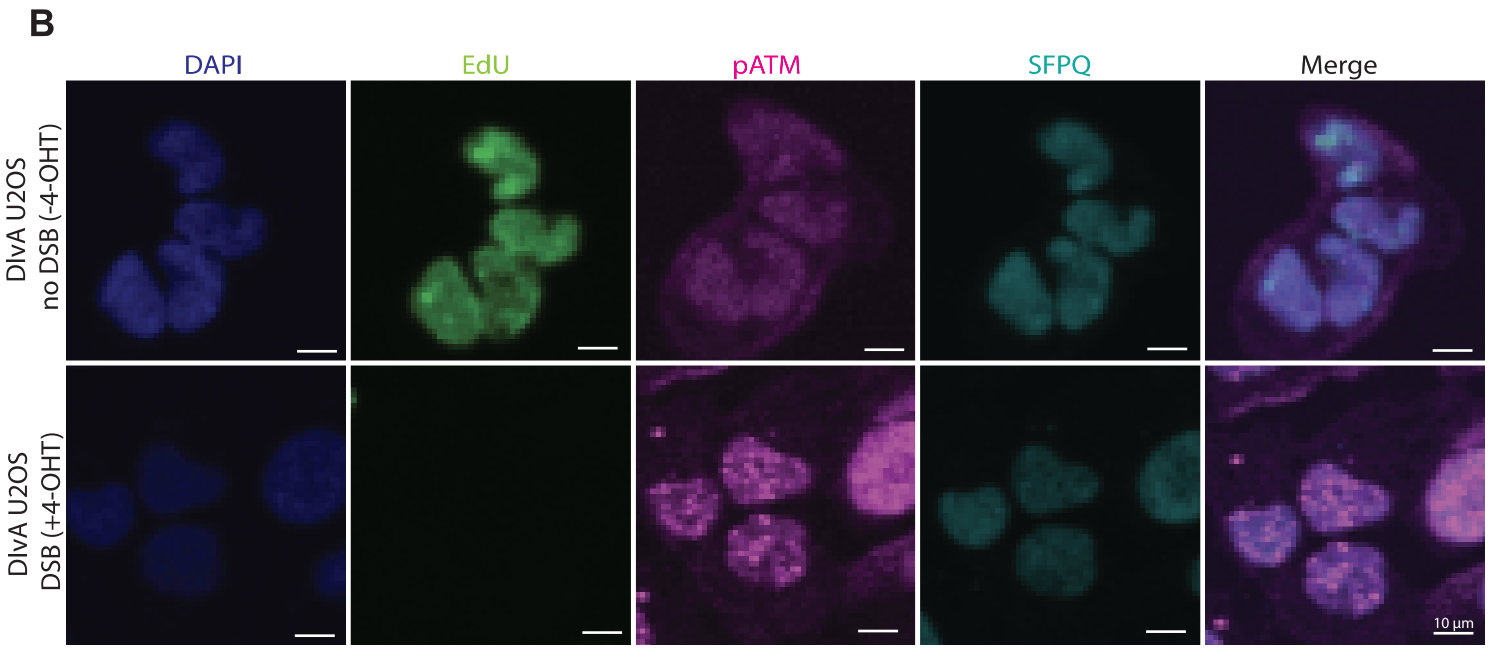

Figure S2

bioRxiv preprint doi: https://doi.org/10.1101/2025.09.08.674956; this version posted September 8, 2025. The copyright holder for this preprint (which was not certified by peer review) is the author/funder, who has granted bioRxiv a license to display the preprint in perpetuity. It is made available under aCC-BY 4.0 International license.

A

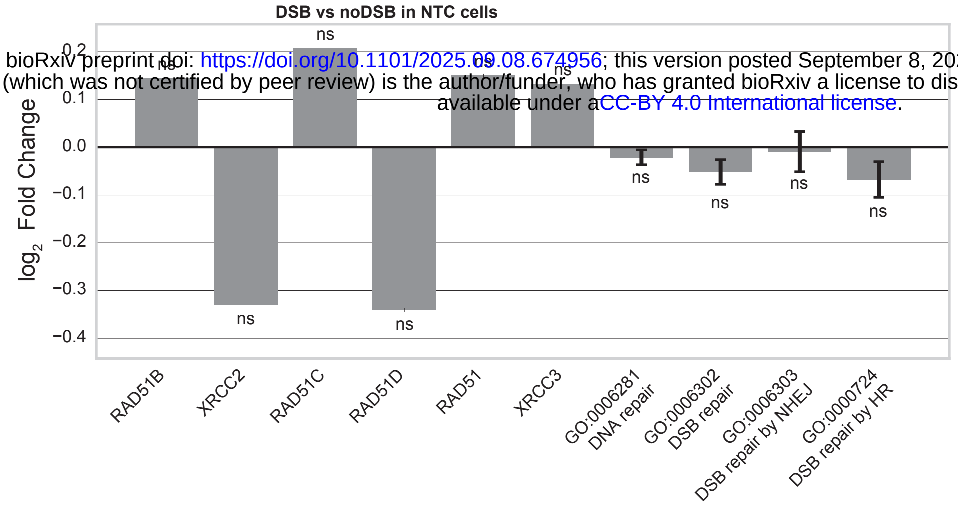

B

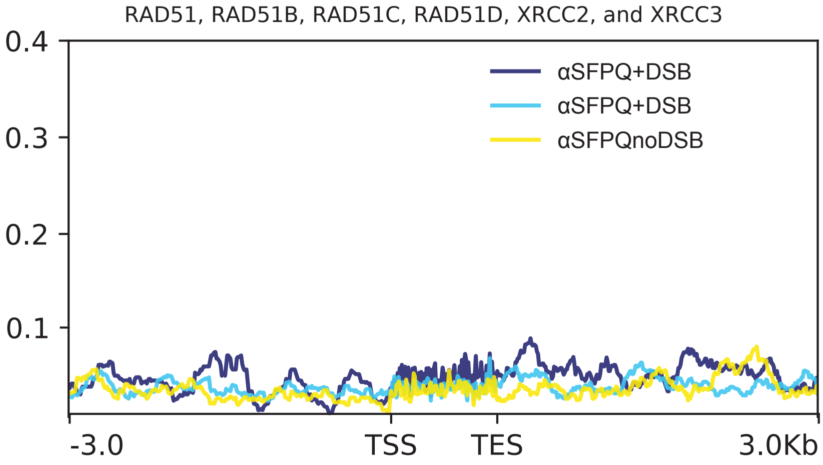

C

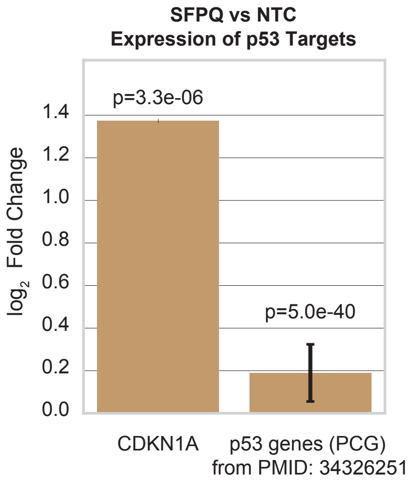

D

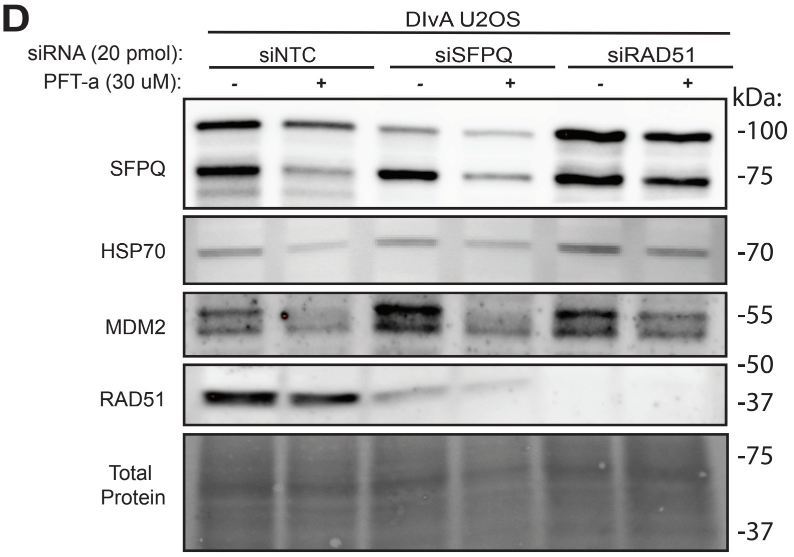

E

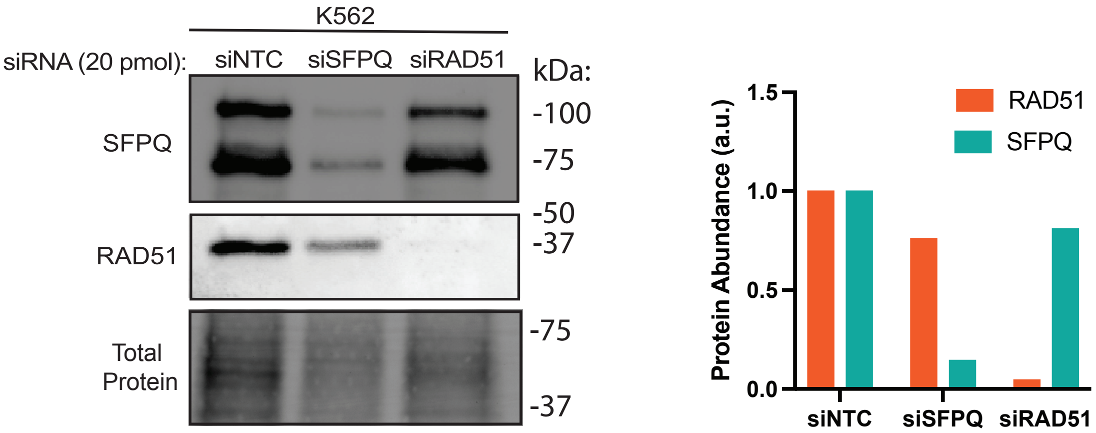

Figure S3

A

bioRxiv preprint doi: https://doi.org/10.1101/2025.09.08.674956; this version posted September 8, 2025. The copyright holder for this preprint (which was not certified by peer review) is the author/funder, who has granted bioRxiv a license to display the preprint in perpetuity. It is made available under aCC-BY 4.0 International license.

RAD51 protein stability Replicate 2

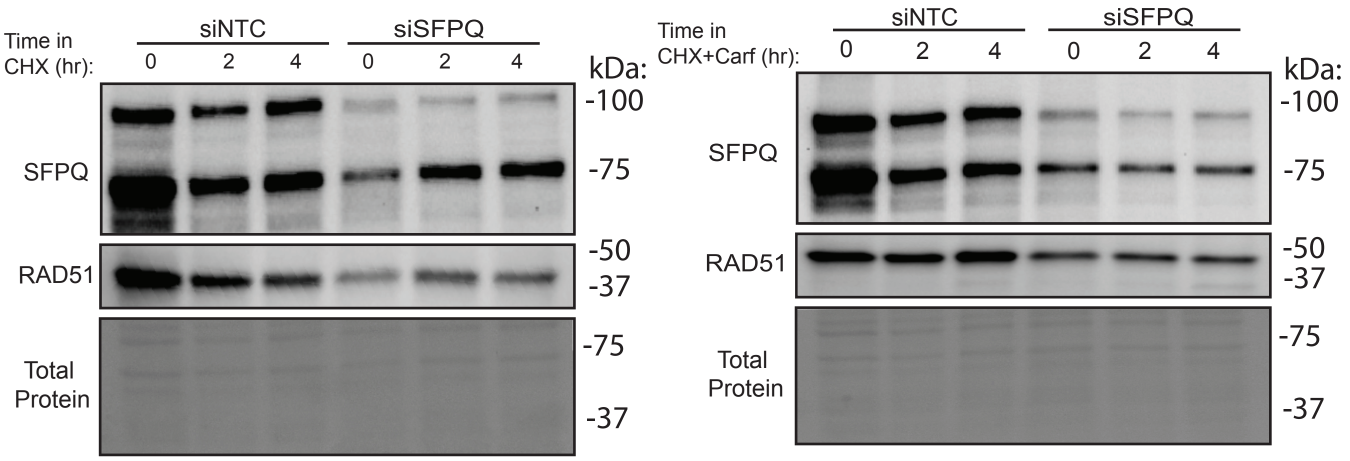

RAD51 protein stability Replicate 3

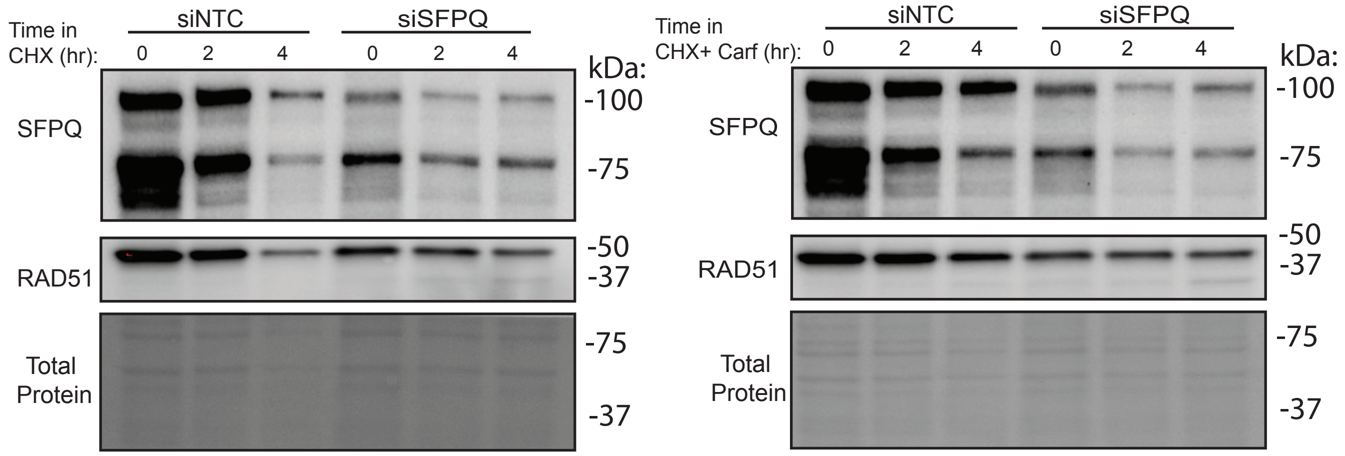

Figure S4

bioRxiv preprint doi: https://doi.org/10.1101/2025.09.08.674956; this version posted September 8, 2025. The copyright holder for this preprint (which was not certified by peer review) is the author/funder, who has granted bioRxiv a license to display the preprint in perpetuity. It is made available under aCC-BY 4.0 International license.

A

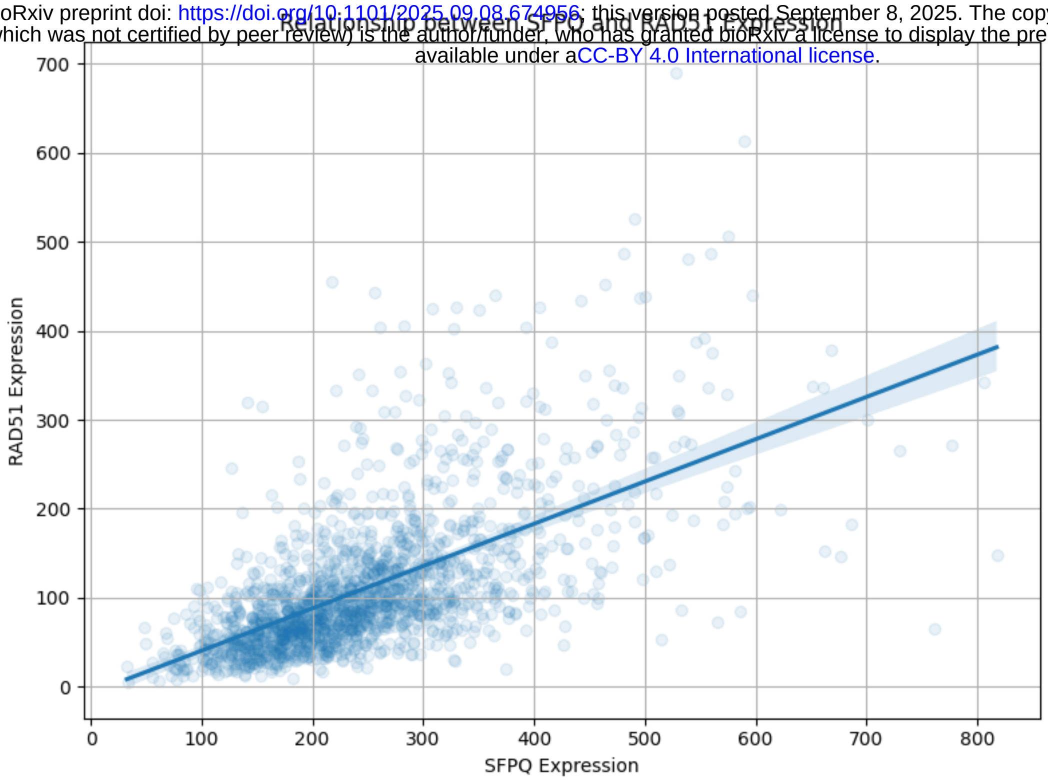

Pearson correlation: 0.625 (p=6.88e-188)
